# Influence of the sickle cell trait on *Plasmodium falciparum* infectivity from naturally infected gametocyte carriers

**DOI:** 10.1186/s12879-023-08134-x

**Published:** 2023-05-10

**Authors:** Christelle M. Ngou, Albert N. Bayibéki, Luc Abate, Olesula S. Makinde, Lionel B. Feufack-Donfack, Elangwe M. Sarah-Matio, Aline G. Bouopda-Tuedom, Paul Taconet, Nicolas Moiroux, Parfait H. Awono-Ambéné, Arthur Talman, Lawrence S. Ayong, Antoine Berry, Sandrine E. Nsango, Isabelle Morlais

**Affiliations:** 1 Institut de Recherche pour le Développement, MIVEGEC, Univ. Montpellier, CNRS, IRD, 91 Avenue Agropolis, BP 64501, 34394 Montpellier, France; 2 Malaria Research Unit, Centre Pasteur du Cameroun, Yaoundé, Cameroon; 3 Université Catholique d’Afrique Centrale, Yaoundé, Cameroon; 4 Department of Statistics, Federal University of Technology, P.M.B 704, Akure, Nigeria; 5 Institute for Parasitology, Centre for Infection Medicine, University of Veterinary Medicine Hannover, Hannover, Germany; 6 Department of Biological Sciences, Faculté de Médecine et des Sciences Pharmaceutiques, Université de Douala, Douala, Cameroon; 7 Laboratoire d’Entomologie Médicale, OCEAC, BP288 Yaoundé, Cameroon; 8 Institut Toulousain des Maladies Infectieuses et Inflammatoires (Infinity), Université Toulouse, CNRS UMR5051, INSERM UMR1291, UPS, Toulouse, France; 9 Service de Parasitologie_Mycologie, Centre Hospitalier Universitaire de Toulouse, Toulouse, France

**Keywords:** Sickle cell trait, Asymptomatic carriers, Malaria transmission, Mosquito infectivity, Gametocytes

## Abstract

**Background:**

Sickle cell trait (SCT) refers to the carriage of one abnormal copy of the β-globin gene, the HbS allele. SCT offers protection against malaria, controlling parasite density and preventing progression to symptomatic malaria. However, it remains unclear whether SCT also affects transmission stages and mosquito infection parameters. Deciphering the impact of the SCT on human to mosquito malaria transmission is key to understanding mechanisms that maintain the trait in malaria endemic areas.

**Methods:**

The study was conducted from June to July 2017 among asymptomatic children living in the locality of Mfou, Cameroon. Blood samples were collected from asymptomatic children to perform malaria diagnosis by microscopy, *Plasmodium* species by PCR and hemoglobin typing by RFLP. Infectiousness of gametocytes to mosquitoes was assessed by membrane feeding assays using blood from gametocyte carriers of HbAA and HbAS genotypes. A zero-inflated model was fitted to predict distribution of oocysts in mosquitoes according to hemoglobin genotype of the gametocyte source.

**Results:**

Among the 1557 children enrolled in the study, 314 (20.16%) were of the HbAS genotype. The prevalence of children with *P. falciparum* gametocytes was 18.47% in HbAS individuals and 13.57% in HbAA, and the difference is significant (χ^2^ = 4.61, *P* = 0.032). Multiplicity of infection was lower in HbAS gametocyte carriers (median = 2 genotypes/carrier in HbAS versus 3.5 genotypes/carrier in HbAA, Wilcoxon sum rank test = 188, *P* = 0.032). Gametocyte densities in the blood donor significantly influenced mosquito infection prevalence in both HbAS and HbAA individuals. The HbAS genotype had no significant effect on mosquito infection outcomes when using immune or naïve serum in feeding assays. In AB replacement feeding experiments, the odds ratio of mosquito infection for HbAA blood as compared to HbAS was 0.56 (95% CI 0.29–1.10), indicating a twice higher risk of infection in mosquitoes fed on gametocyte-containing blood of HbAS genotype.

**Conclusion:**

Plasmodium transmission stages were more prevalent in SCT individuals. This may reflect the parasite’s enhanced investment in the sexual stage to increase their survival rate when asexual replication is impeded. The public health impact of our results points the need for intensive malaria control interventions in areas with high prevalence of HbAS. The similar infection parameters in feeding experiments where mosquitoes received the original serum from the blood donor indicated that immune responses to gametocyte surface proteins occur in both HbAS and HbAA individuals. The higher risk of infection in mosquitoes fed on HbAS blood depleted of immune factors suggests that changes in the membrane properties in HbAS erythrocytes may impact on the maturation process of gametocytes within circulating red blood cells.

**Supplementary Information:**

The online version contains supplementary material available at 10.1186/s12879-023-08134-x.

## Background

Malaria parasites have exerted the strongest known selective pressure on the human genome over the last 10,000 years [1]. Polymorphisms in red blood cell (RBC) genes that confer a protective role against *Plasmodium falciparum* malaria have been maintained in the human population and their highest frequencies have been found in sub-Saharan Africa [1, 2]. Sickle cell disease (SCD) is among the most common monogenic disorders worldwide. It results from a single amino acid substitution in the beta globin protein leading to abnormal hemoglobin S (HbS). Heterozygous individuals (HbAS) of this allele have sickle cell trait (SCT) and do not have symptoms of SCD, whilst homozygous individuals (HbSS) suffer from sickle cell anemia (SCA) and majority of SCA children died before the age of 5 years [3].

SCT protects against severe and uncomplicated malaria, which is linked to diminished proliferation of the parasite asexual stage, and lower malaria parasite densities are reported in febrile cases [4–6]. Protection against symptomatic malaria has specifically been associated with several mechanisms including but not limited to growth inhibition in low oxygen conditions [7], impaired cytoadherence [8] or altered hemoglobin digestion [9]. By contrast, studies that investigated the protection of SCT against asymptomatic *P. falciparum* infection have reported inconsistent results [10–12]. SCT was shown to reduce the frequency of asymptomatic infections and delay the onset of malaria [5, 11] while no association between malaria asymptomatic carriage and hemoglobin genotype was found in other reports [10, 12].

Asymptomatic malaria infections represent an important reservoir for parasites and contribute to onward mosquito infections [13]. In endemic areas, malaria infections generally carry multiple genotypes of *P. falciparum* [14, 15]. Previous studies have evidenced that the genetic diversity within the gametocyte populations influences the transmission dynamics [16–18]. Monoclonal infections led to higher oocyst loads in the mosquito [17]. In polyclonal infections, parasites have to face their conspecifics and competitive interactions between co-infecting genotypes modulate the intra-host dynamics of parasites [19, 20]. Our previous works suggested that minority genotypes would subvert immune responses to other parasites in order to optimize their transmission by the vector [17, 18].

Transmission from human to mosquito relies on the availability of sexual stages, the gametocytes, and multiple factors such as gametocyte density, sex ratio, maturity or other human and mosquito factors known to influence parasite transmissibility [13, 21]. It has been previously reported that human genetic factors contribute to gametocyte prevalence in asymptomatic infections and the HbS mutation was found associated with increased gametocyte positivity in Senegal [22]. Earlier work had also evidenced that individuals carrying genetic variants of the HBB gene resulted in an enhanced infectivity to mosquitoes. Indeed, mosquitoes fed on gametocyte-containing blood from HbAS and HbAC individuals gave rise to higher infection rates as compared to those fed on blood from HbAA individuals, despite similar gametocyte densities [23, 24].

These studies have not elucidated how this enhanced infectivity may occur and what other infection parameters may be associated with SCT and transmission. Here, we investigate the association between malaria transmission parameters and SCT among asymptomatic children in an area of high malaria transmission in Cameroon.

## Methods

### Ethics approval and consent to participate

This study was conducted in accordance with the principles of the Declaration of Helsinki and was approved by the National Committee of Ethics Research for Human Health (CNERSH) under agreement 2017/06/922/CE/CNERSH/SP and obtained research agreement from the Ministry of Health (032/L/MINSANTE/SG/DRSPC/SSD/MFOU). The informed consent was obtained from legal parent/guardian. All participants were enrolled as volunteers after their legal parent/guardian signed the informed consent form.

### Study population and collection of blood samples

Screening surveys were conducted in June 2017 in villages of the health district of Mfou, about 30 km from Yaoundé, Cameroon. Recruitment was performed without knowledge of the genetic background of volunteers. Malaria diagnosis was performed upon microscopic examination of Giemsa-stained blood smears. Parasite-positive volunteers received free treatment with Artesunate Amodiaquine according to national guidelines. Parasite densities were estimated per microliter of blood, by counting the number of trophozoites per 500 leucocytes and gametocytes per 1000 leukocytes, and assuming a standard number of 8000 leukocytes/µL. Dried blood spots (DBSs) on Whatman^®^ Grade 17 paper were obtained from finger pricks. Genomic DNA from DBSs was extracted using the Chelex-100 (Bio-Rad Laboratories, USA) method as previously described [25]. DNA extracts were used for molecular detection of *Plasmodium* spp using a multiplex PCR as described in Padley [26] and for hemoglobin genotyping by PCR–RFLP according to Saiki and colleagues [27]. Venous blood samples were collected from volunteers carrying *P. falciparum* gametocytes. A 5 ml heparinised tube was used for experimental infections of mosquitoes and a 5 ml EDTA tube for gametocyte purification and *P. falciparum* genotyping.

### Gametocyte purification and *P. falciparum* genotyping

Gametocyte purification was performed using a MACS^®^ system (Miltenyi Biotec, Bergisch Gladbach, Germany) with magnetic columns using 1 ml of serum-free blood as previously described [28]. Genotyping was performed at seven microsatellite loci according to Anderson et al. [29]. Labelled PCR products were analysed on an ABI 3500 XL DNA genetic analyser (Applied Biosystems, Foster City, CA) at the GenSeq platform in Montpellier and alleles determined with the GeneMapper^®^ software (v6, Applied Biosystems). Multiplicity of infection (MOI) was defined as the maximum number of alleles at the most polymorphic locus.

### Membrane feeding assays and mosquito infections

Membrane feeding assays (MFAs) were performed using the local Ngousso colony of *An. coluzzii*, as previously described [17, 30]. For each gametocyte donor, feeding experiments were set under three blood conditions: whole blood (WB) and serum replacements with serum from the same carrier’s (OWN) or naïve AB serum (AB). In WB assays, fresh blood was provided directly to female mosquitoes. In serum replacement assays, the gametocyte carrier plasma was removed after 5 min of centrifugation at 2000 rpm and replaced either by an AB serum from a non-immune European donor (AB) or by an equal volume of the original plasma (OWN). For all MFAs, 400 µl volume of blood was dispensed into pre-warmed glass feeders and batches of 50–80 female mosquitoes starved overnight were allowed to feed for 25 min.

Fully engorged female mosquitoes were kept in the insectary under standard conditions (28 ± 2 °C, 80 ± 5% relative humidity, 12/12-h light/dark cycle) until dissection at 8 days post-feeding. Individual midguts were examined under a light microscope at X200 magnification for oocyst counts. Prevalence of infection was defined as the proportion of mosquitoes with at least one *P. falciparum* oocyst per midgut and intensity of infection as the mean number of oocysts per infected mosquito.

### Data analysis

Statistical analyses were carried out using R (version 4.0.5). For data from the screening surveys among children, the effects of hemoglobin variant, sex, age and school were tested for parasite prevalence and infection densities. Prevalences of infection were compared using χ^2^ test. Trophozoites and gametocytes densities were compared using Wilcoxon sum rank test for two group comparisons on log-transformed parasite densities and generalized linear models (GLMs) were used for multiple comparisons. *P*-values less than 0.05 were considered statistically significant.

Oocyst count data are overdispersed and inflated in zeros. We then applied zero-inflated negative binomial models, as they were the best fitted to our dataset. The predictor variables fitted in the models include hemoglobin status, MOI and gametocyte densities, and the blood donor was set as a random effect to control for variation between individuals. Gametocyte densities and MOI were centered and scaled prior to modeling. In the model, the zero-inflation component contains logit coefficients for predicting excess zeros and the negative binomial regression component estimates the effect of a predictive variable on the count data. Maximum likelihood estimates were obtained using the glmmTMB package in R.

## Results

### Description of the study population

A total of 1557 asymptomatic children aged 1–16 years were enrolled in 12 villages from the Mfou health district. Hemoglobin genotyping identified 1238 (79.51%) individuals of the HbAA genotype, 314 (20.16%) of the HbAS genotype and 5 (0.32%) were of the HbSS genotype. Given the small number of HbSS subjects, they were not included in further analyses. Males were over represented in the HbAS group (57.3%, *P* = 0.0125, Table 1).

**Table 1:** Characteristics of the population

	HbAA (n = 1238)	HbAS (n = 314)	OR [95% CI]	*P*_value
Gender, n (%)				
Male	612 (49.43)	180 (57.32)	1.37 [1.07–1.76]	0.012*
Female	626 (50.57)	134 (42.68)		
Median age [range]	9 [1–16]	8 [1–15]		
Age groups (years), n (%)				
≤ 5	239 (19.31)	64 (20.38)	1.07 [0.79–1.45]	0.667
6–10	567 (45.80)	157 (50)	1.18 [0.92–1.52]	0.183
> 10	432 (34.89)	93 (29.62)	0.785 [0.60–1.02]	0.078

*OR* odds ratio, *CI* confidence interval; *statistically significant with the Chi-squared test

### Malaria distribution in the studied area

Prevalence of *Plasmodium* infection was 68.7% (1066/1552). *P. falciparum* was the most prevalent species, present in 95.3% of the infections (1016/1066), followed by *P. malariae*, identified in 27.8% of the infections (297/1066). *P. ovale* was found in only one infection (Additional file 1: Table S1). Mixed *P. falciparum—P. malariae,* infections were observed with a 23.3% prevalence (248/1066).

We computed results of *P. falciparum* prevalence and parasite densities according to host factors (sex, hemoglobin type, age) or the participant village and data are shown in Additional file 1: Table S2.

**Fig. 1:**
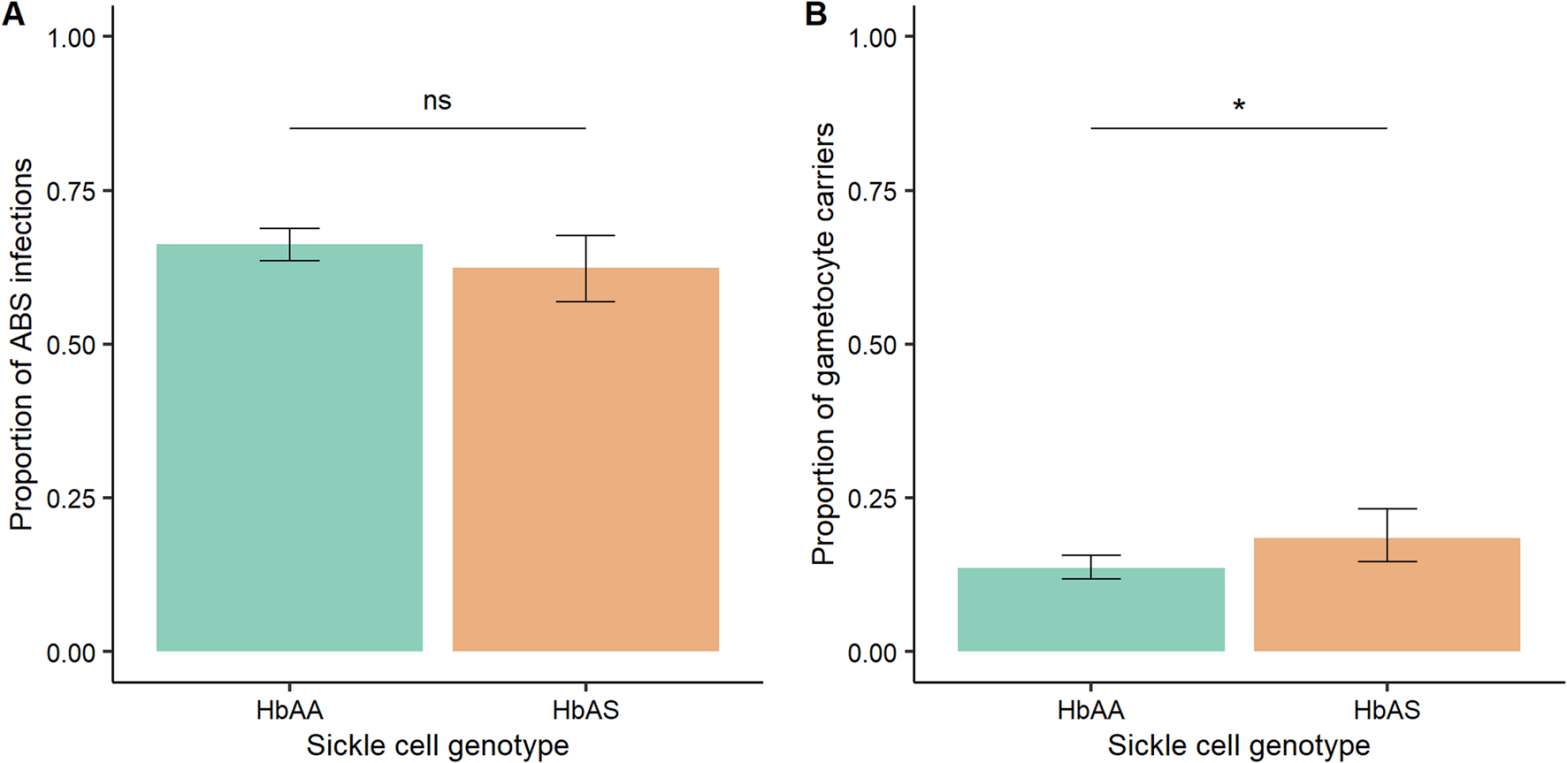
Proportion of *P. falciparum* infection according to sickle cell genotype for asexual blood stages (**A**) and gametocyte stages (**B**). HbAA (green), homozygous for normal hemoglobin; HbAS (orange), heterozygous for hemoglobin S. ABS, asexual blood stages. Significance was assessed by the Chi-squared test; ns, non significant; *P < 0.05. Black bars indicate the standard errors

Prevalence of *P. falciparum* infection in HbAA and HbAS individuals was similar for asexual blood stage (ABS) carriage, 66.24% (820/1238) vs 62.42% (196/314), respectively (χ^2^ = 1.59, *P* = 0.206, Fig. 1A and Additional file 1: Table S2). However, prevalence of *P. falciparum* gametocyte carriage was significantly higher in HbAS individuals, 18.47% (58/314), as compared to HbAA individuals, 13.57% (168/1238) (χ^2^ = 4.61, *P* = 0.032**,** Fig. 1B and Additional file 1: Table S2). Parasite densities did not differ between HbAA and HbAS groups, neither for asexual (Wilcoxon rank sum test statistic = 78232, *P* = 0.564) or gametocyte stages (Wilcoxon rank sum test statistic = 4934.4, *P* = 0.874) (Additional file 1: Table S2).

### Characteristics of the *P. falciparum* gametocyte carriers used for membrane feeding assays

**Fig. 2:**
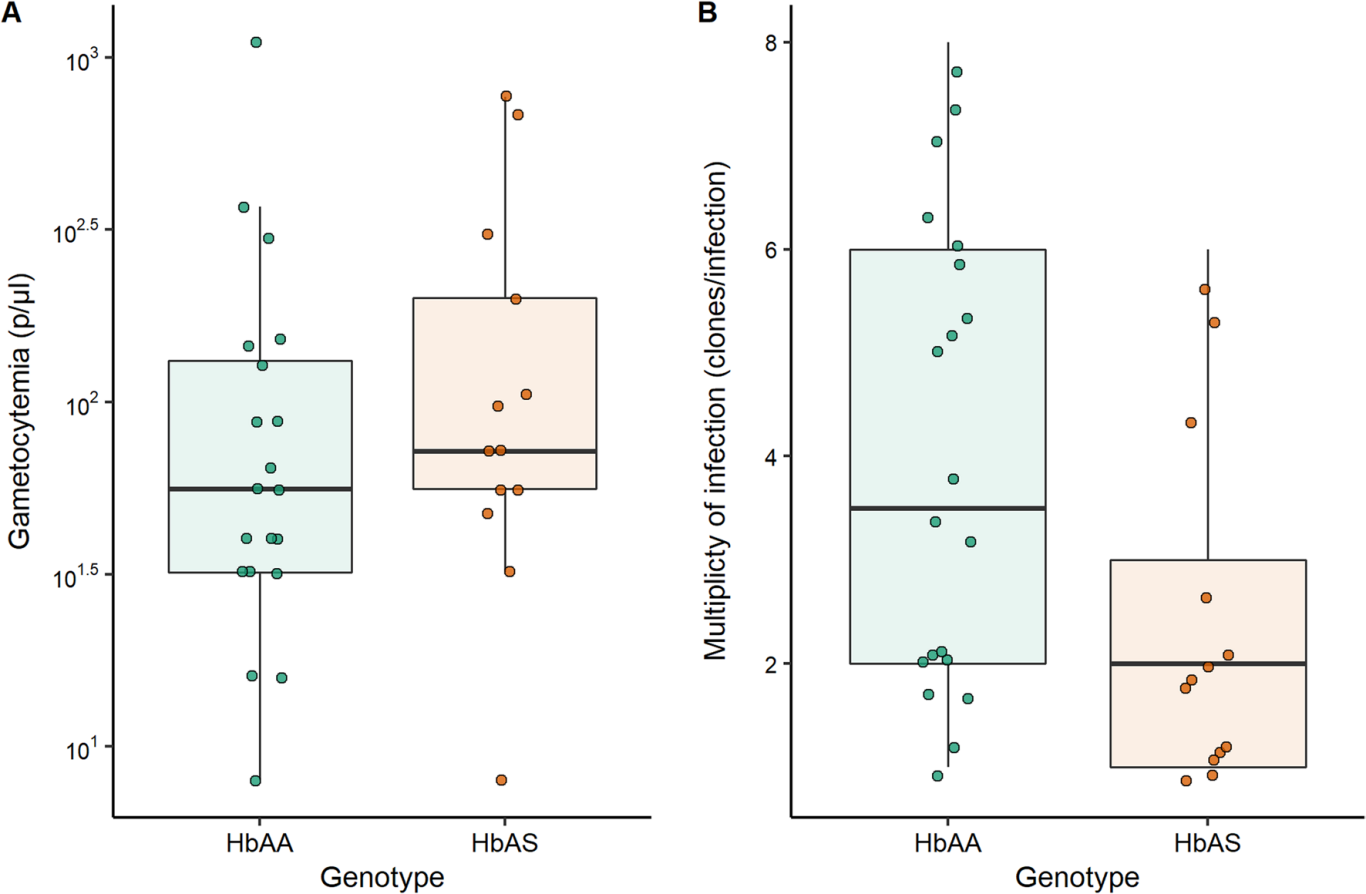
Parasitological data of HbAA (green) and HbAS (orange) gametocyte carriers used for membrane feeding assays. **A** median gametocyte densities in HbAS, 72 parasites/µl [IQR 56–200], and in HbAA, 56 parasites/µl [IQR 32–132], were not different (Wilcoxon sum rank test = 1101, *P* = 0.292*)*. **B** MOI of gametocyte stages in HbAS carriers was significantly lower than that in their HbAA counterparts, 2 [IQR 1–3] versus 3.5 [IQR 2–6] respectively (Wilcoxon sum rank test = 188, *P* = 0.032). Each dot represents data from a gametocyte donor

We selected *P. falciparum* gametocyte positive children among the participants for membrane feedings. A total of 34 gametocyte carriers aged from 3 to 13 years were included for ex vivo transmission assays (Additional file 1: Table S3). Among them, 21 individuals were of the HbAA genotype and 13 were of the HbAS genotype. Gametocyte densities were 20% higher in the HbAS cohort as compared to the HbAA parasite donor, however the difference did not reach statistical significance (W = 101, *P* = 0.292, Fig. 2A). Gametocyte carriers of HbAS group had significantly fewer *P. falciparum* genotypes as compared to the HbAA group (HbAS, median MOI = 2 [IQR 1–3] and HbAA, median MOI = 3.5 [IQR 2–6]; W = 188, *P* = 0.032 and Fig. 2B).

### Infectivity of gametocyte carriers to mosquitoes

**Fig. 3:**
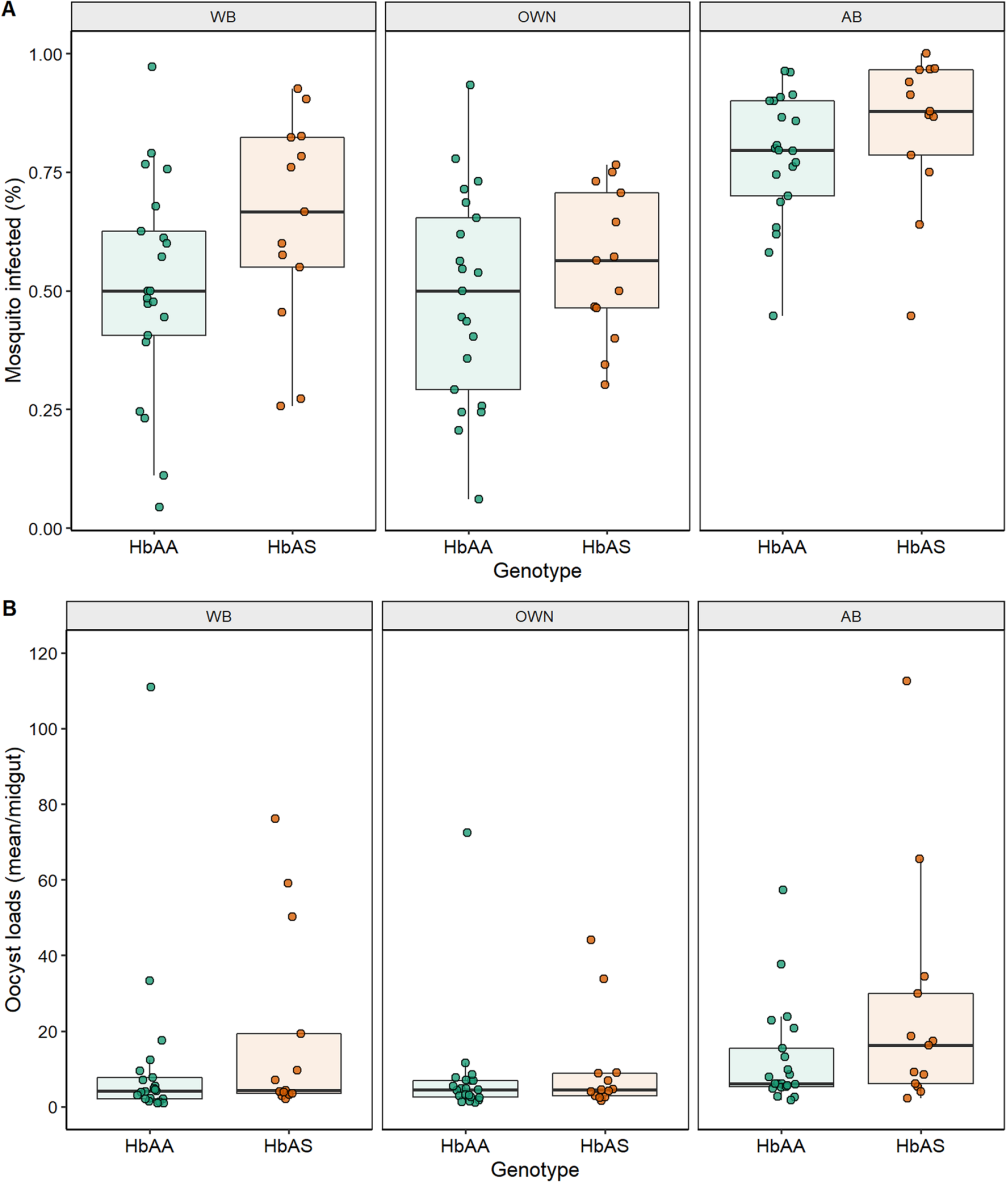
Infectivity of HbAA (green) and HbAS (orange) gametocyte carriers to mosquitoes in the different membrane feeding assays. **A** percentage of infected mosquitoes and **B** mean oocyst densities in infected mosquitoes. WB, membrane feeding assays using whole blood. OWN, blood mixture with the serum from the donor. AB, membrane feeding assays with naïve serum replacement. Each dot represents data from a gametocyte donor

Mosquito infection prevalence and oocyst intensity for each blood donor and for the three membrane feeding experiments, whole blood, OWN and AB are presented in Additional file 1: Table S3. Mosquito infection parameters according to the HBB genotype for the three feeding conditions are illustrated in Fig. 3.

Estimates from the zero-inflated negative binomial GLMM were computed for the three blood feeding conditions (Table 2, Additional file 1: Table S4 and S5). The count model provided estimates of the effect of the predictive variables (HBB genotype, gametocyte density, and MOI) on oocyst counts with incidence rate ratio, confidence interval (IC) and *P*-value. The zero-inflation model contains estimates for predicting excess zeros with the corresponding statistics, odd ratio (OR), CI and *P*-values.

**Table 2:** Estimates of zero-inflated negative binomial generalized linear mixed model for oocysts count of AB feedings

Count model			
Predictors	IRR	CI	*P*-value
(Intercept)	9.18	6.47–13.02	**< 0.001**
genotype [HbAS]	1.36	0.77–2.40	0.296
gametocytemia	2.00	1.52–2.62	**< 0.001**
MOI	0.99	0.74–1.33	0.955

Zero-inflation model			
Predictors	OR	CI	*P*-value
(Intercept)	0.22	0.14–0.33	**< 0.001**
genotype [HbAS]	0.66	0.33–1.33	0.250
gametocytemia	0.66	0.44–0.98	**0.037**
MOI	1.03	0.73–1.44	0.868

Bold values denote statistical significance at the *P* < 0.05 level

Dispersion parameter for truncated_nbinom2 family (): 1.95; MOI, multiplicity of infection; IRR, Incidence Rate Ratio; OR, Odd Ratio; CI, confidence interval

**Fig. 4:**
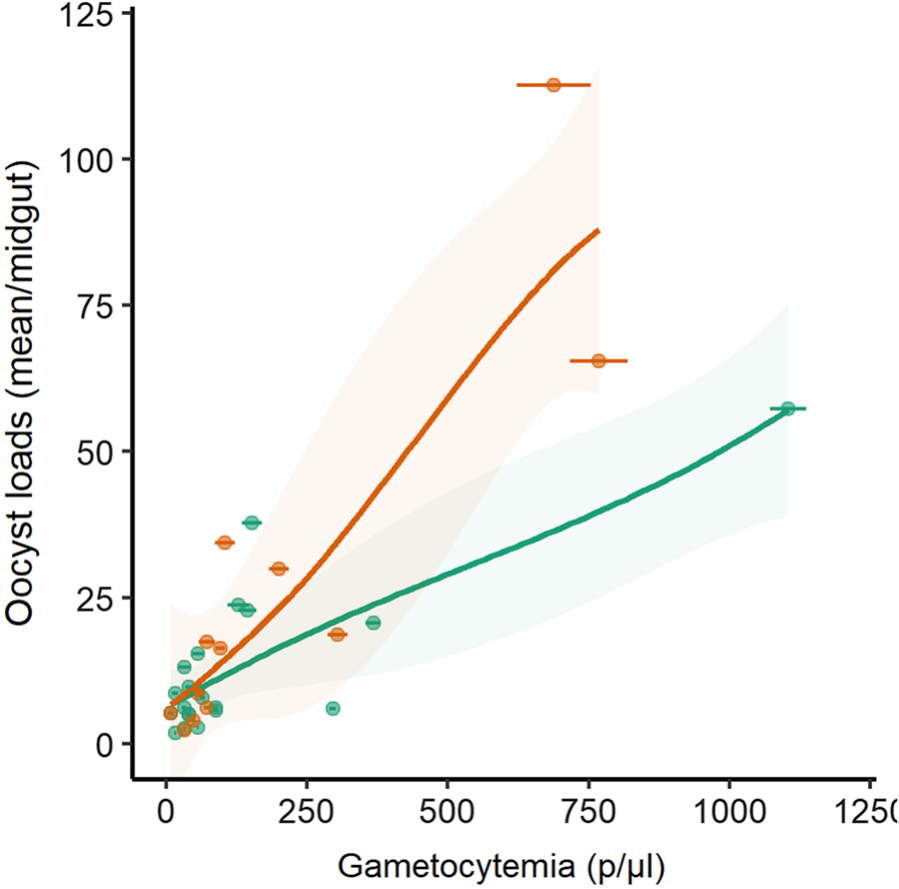
Mosquito infection burden according to gametocytemia in HbAA and HbAS blood donors. The relationship between gametocyte density of the blood donor and oocyst intensity was assessed for HbAA (green) and HbAS (orange) individuals. The difference in oocyst loads between mosquitoes fed on HbAA and HbAS blood increased with gametocyte densities but did not reach significant level (Wald test, *P* = 0.318)

Correlations of the number of oocysts with gametocyte density were significant for the three feeding conditions (count models in Table 2, Additional file 1: Table S4 and Table S5). Hence, oocyst count is associated with gametocyte density in the blood donor for each feeding condition, whole blood, OWN and AB. In addition, the HBB genotype was a non-significant factor in the count models, indicating that mean oocyst loads in mosquitoes increased significantly with increasing gametocyte density regardless of the HBB genotype. Similarly, the odds of excess zeros (zero-inflation model) decreased with increasing gametocyte density and HBB genotype was a non-significant factor in the zero-inflation models. This indicates that mosquitoes are more likely to have any oocysts as gametocyte density increases in the blood donor, for both HBB genotypes. This relation between gametocyte density and mosquito infection prevalence was significant only in AB replacement experiments (Table 2). We then tested the models with the HBB genotype as a single predictor variable and results confirmed that the HBB genotype did not have significant effect on mosquito prevalence or oocyst loads (Additional file 1: Table S6, S7 and S8). In AB replacement feeding experiments, the odds ratio of mosquito infection for HbAA blood as compared to HbAS was 0.56 (95% CI 0.29–1.10), indicating a twice higher risk of infection in mosquitoes fed on gametocyte-containing blood of HbAS genotype. And although the HbAS genotype was associated with higher log oocyst counts in mosquitoes as compared to HbAA genotype at the highest gametocyte densities, the difference did not reach significant level (Wald test, *P* = 0.318, Fig. 4).

## Discussion

In this study, hemoglobin genotyping was performed on blood samples collected from children attending primary schools in the malaria endemic area of Mfou. Mosquitoes were infected with blood from HbAS and HbAA participants identified with *P. falciparum* gametocytes to investigate the effect of the hemoglobin genotype on parasite infectivity.

In our studied area, about two third of the children (65.5%) presented *Plasmodium* infection and 95% of the infections harboured *P. falciparum,* in line with our previous works in the same locality of Mfou [31–33]. *P. falciparum* prevalence has even increased over the past 15 years, from 51% in 2005–2006, 55% in 2013–2014 to 66% in 2017 and 2018, despite continued control interventions.

Prevalence of SCT was 20.2% in our cohort, which is in agreement with the reported range in Central Africa [2, 34, 35]. In our study, malaria prevalence and *P. falciparum* asexual densities did not differ significantly between the HbAS and HbAA groups, which is in agreement with other previous surveys [6, 36, 37]. Few studies have analysed the impact of SCT on asymptomatic individuals and these works were conducted in areas with varying epidemiological settings, making it difficult to draw consistent conclusion on the protective effect of HbAS on asymptomatic malaria. Nonetheless, SCT could provide indirect protection that is likely missed in cross-sectional studies, particularly in high transmission settings where children develop acquired antimalarial immunity by the age of 5. Indeed, longitudinal studies in malaria endemic areas have provided evidence that HbAS delayed both time to first malaria episode and parasite reappearance after antimalarial treatment [5, 11, 37, 38]. In our study, we found a higher prevalence of *P. falciparum* gametocytes in individuals with HbAS, which corroborates a former work from Uganda [5]. In this later study, the authors suggested that the longer duration of asymptomatic infections in HbAS children would explain the higher prevalence of *P. falciparum* gametocytes in SCT carriers [5]. Altered immune responses to gametocytes in HbAS could also contribute to limit clearance of RBCs infected with gametocytes [39, 40].

The lower multiplicity of infection we found in gametocyte samples of HbAS genotypes likely results from lower MOI in asexual stages. Previous studies that investigated parasite genetic diversity in SCT carriers reported contrasting results. Gong et al. reported lower MOI in blood samples from HbAS children while Ntoumi and colleagues found a higher prevalence of polyclonal infections in SCT children [5, 35]. Differences in transmission intensity, age groups and the small sample sizes probably explain these discrepancies. The protective effect of SCT against symptomatic malaria that limits parasite growth may impede the establishment of co-infecting strains of parasites [5, 6] and this would explain the trend for larger monoclonal infections in HbAS. Interestingly, a recent study from human blood collections reports an association between HbS and parasite polymorphisms in three genes [41]. It will be important to genotype these loci also in gametocyte and oocyst transmission stages to determine if they are found at all parasite developmental stages and to understand what the fitness advantage/cost of these mutations may be in HbAS and HbAA individuals.

In this study, we found that the risk of infection was doubled for mosquitoes fed on HbAS blood when compared to those fed on HbAA blood in the serum replacement feeding condition, and this result is consistent with previous studies [23, 24]. Furthermore, although not significant, we observed that the oocyst load tends to increase faster with increasing gametocyte densities in mosquitoes fed on HbAS blood. These results may reflect a higher fitness of gametocytes in an HbAS background. A higher risk of mosquito infection in HbAS could be linked to a longer period of circulation of mature stage V gametocytes in HbAS blood, which would provide parasites a greater chance to reach the peak of infectivity for the mosquito. Altered membrane properties of HbAS erythrocytes were recently described [42] and these changes may impact the maturation process of gametocytes within red blood cells. Gametocyte density in the blood donor was the only variable that statistically influenced mosquito prevalence and intensity in our model, for both HbAA and HbAS blood. While it is acknowledged that gametocyte density is the main determinant of mosquito infection outcomes, other parameters such as gametocyte sex-ratio, genetic diversity or immune factors also affect mosquito infectivity [16, 43–45]. The lower MOI we found in HbAS blood donors likely limits the amount of outcrossing. We have previously reported that mating between clone relatives results in higher mosquito infections [16], monoclonal infections reaching the highest infection rates. Other works also reported restrained outbreeding amongst malaria parasites in areas of intense transmission [20, 46].

The increased risk of infection for mosquitoes fed on HbAS blood was not evidenced in membrane feedings where naturally acquired antibodies were present (OWN). This indicates that both HbAS and HbAA individuals are capable of mounting immune responses against gametocyte infected RBCs with an equivalent transmission reducing activity. Human antibody responses to surface gametocyte proteins are known to reduce infectiousness of the sexual stage in the mosquito and this was the basis for the development of transmission blocking vaccines [47–49]. Thus, our data suggest that malaria transmission blocking vaccines that target antigens expressed at the surface of the parasite sexual stages and induce potent antibodies should have a same efficiency in HbAS and HbAA individuals.

A limitation of our study is the small sample size for each of the HBB genotypes, HbAA and HbAS, used for membrane feeding assays and larger assays will be necessary to accurately determine if the HBB genotype has an impact on gametocyte infectiousness. Additionally, in our membrane feeding assays, gametocyte donor children were aged over 5 years and the observed effect may vary in other age groups. Indeed, previous studies have reported that protection of HbAS against symptomatic malaria or high density parasitemia varied with age as a result of acquired immunity in endemic areas [4–6, 11].

## Conclusion

Our study indicated a higher risk of mosquito infection when fed on blood from HbAS individuals and this suggests higher infectiousness of gametocytes circulating in HbAS blood. Our observation may represent a mechanism underlying host–pathogen co-evolution, whereby *P. falciparum* would have developed means to increase its infectiousness for the mosquito to compensate the protection against malaria conferred by the SCT in the human host. In HbAS individuals, protection against severe malaria occurs through reduced cytoadherence capacity of infected RBCs to microvascular blood vessel endothelium and increased oxidative stress in RBCs infected by asexual parasites [8, 50, 51]. However, so far, no study has investigated the molecular and biochemical changes caused by the HbS carriage on gametocytogenesis. Further studies will have to address whether gametocyte-infected HbAS cells harbour different properties that could affect maturity and infectiousness. Nonetheless, the public health impact of our results points the need for intensive malaria control interventions in areas with high prevalence of HbAS.

## Supplementary Information

**Additional file 1: Table S1.** Distribution of Plasmodium infections in the cohort and according to the hemoglobin type of the children. **Table S2.** Parasite metrics of the participants. **Table S3.** Characteristics of the gametocyte donors and parameters of mosquito infection according to the feeding experiments. **Table S4.** Estimates of the zero-inflated negative binomial generalized linear mixed model for oocyst count in the OWN membrane feeding condition assays. **Table S5.** Estimates of the zero-inflated negative binomial generalized linear mixed model for oocyst count in the whole blood membrane feeding assays. **Table S6.** Results of the zero-inflated negative binomial generalized linear mixed model for oocyst count in the AB replacement feedings. **Table S7.** Results of the zero-inflated negative binomial generalized linear mixed model for oocyst count in the OWN feeding assays. **Table S8.** Results of the zero-inflated negative binomial generalized linear mixed model for oocyst count in the whole blood feedings.

## Data Availability

The datasets supporting the conclusions of this article are included within the article and its additional files.

## Abbreviations

SCT: Sickle cell trait
HBB: Hemoglobin
HbS: Sickle cell hemoglobin
RBC: Red blood cell
DBS: Dried blood spot
MOI: Multiplicity of infection
MFA: Membrane feeding assay
WB: Whole blood
AB: Naïve serum from a AB donor
OWN: Serum from the donor
CNERSH: National Committee of Ethics Research for Human Health
GLM: Generalized linear models
