# Supplementary material for: Influence of the sickle cell trait on Plasmodium falciparum infectivity from naturally infected gametocyte carriers
Source: BMC Infect Dis. 2023 May 10;23:317. doi: 10.1186/s12879-023-08134-x (PMC10173526; doi:10.1186/s12879-023-08134-x)
Supplement: Supplementary file 1 — Additional file 1: Table S1. Distribution of Plasmodium infections in the cohort and according to the hemoglobin type of the children. Table S2. Parasite metrics of the participants. Table S3. Characteristics of the gametocyte donors and parameters of mosquito infection according to the feeding experiments. Table S4. Estimates of the zero-inflated negative binomial generalized linear mixed model for oocyst count in the OWN membrane feeding condition assays. Table S5. Estimates of the zero-inflated negative binomial generalized linear mixed model for oocyst count in the whole blood membrane feeding assays. Table S6. Results of the zero-inflated negative binomial generalized linear mixed model for oocyst count in the AB replacement feedings. Table S7. Results of the zero-inflated negative binomial generalized linear mixed model for oocyst count in the OWN feeding assays. Table S8. Results of the zero-inflated negative binomial generalized linear mixed model for oocyst count in the whole blood feedings. [file 12879_2023_8134_MOESM1_ESM.docx]

**Table S1** Distribution of Plasmodium infections in the cohort and according to the hemoglobin type of the children

| Plasmodiumspecies | All the cohort | HbAA | HbAS | *P*_value |
| --- | --- | --- | --- | --- |
| *P. falciparum* | 72.05 (768/1066) | 72.33 (622/860) | 70.87 (146/206) | 0.667 |
| *P. malariae* | 4.59 (49/1066) | 4.53 (39/860) | 4.86 (10/206) | 0.853 |
| mixed *P. falciparum / P. malariae* | 23.27 (248/1066) | 23.02 (198/860) | 24.27 (50/206) | 0.714 |
| *P. ovale* | 0.09 (1/1066) | 0.12 (1/860) | 0 (0/206) | na |

**Table S2** Parasite metrics of the participants

| Variable |  | **Asexual blood stages** | | | | **Gametocyte stages** | | | |
| --- | --- | --- | --- | --- | --- | --- | --- | --- | --- |
|  | Category | Prevalence  (n/N) | *P*_value | Density  (sd) | *P*_value | Prevalence  (n/N) | *P*_value | Density  (sd) | *P*_value |
| Sex |  |  | ***0.012*** |  | *0.865* |  | *0.501* |  | *0.539* |
|  | Female | 62.37 (474/760) |  | 3488.88 (8804.55) |  | 13.95 (106/760) |  | 180.83 (1010.39) |  |
|  | Male | 68.43 (542/792) |  | 3623.88 (9644.74) |  | 15.15 (120/792) |  | 54.14 (366.45) |  |
| Hemoglobin | |  | *0.206* |  | *0.564* |  | ***0.032*** |  | *0.874* |
|  | HbAA | 66.24 (820/1238) |  | 3660.12 (9568.52) |  | 13.57 (168/1238) |  | 118.62 (804.37) |  |
|  | HbAS | 62.42 (196/314) |  | 3145.79 (7834.63) |  | 18.47 (58/314) |  | 98.87 (529.98) |  |
| Age class | |  | ***0.004*** |  | ***0.000*** |  | ***0.003*** |  | *0.255* |
|  | ≤ 5 | 65.68 (199/303) |  | 7575.72 (16322.45) |  | 20.79 (63/303) |  | 98.54 (509.27) |  |
|  | 6-10 | 69.09 (502/724) |  | 3013.28 (6877.46) |  | 13.74 (100/724) |  | 183.84 (1039.04) |  |
|  | > 10 | 61.01 (315/525) |  | 1883.19 (4462.98) |  | 11.98 (63/525) |  | 17.02 (18.43) |  |
| Village |  |  | ***0.000*** |  | *0.094* |  | *0.072* |  | *0.091* |
|  | Abembe | 79.79 (79/99) |  | 3508.86 (4679.77) |  | 22.22 (22/99) |  | 22.91 (14.75) |  |
|  | Ekali I | 71.93 (82/114) |  | 2776.59 (10698.78) |  | 17.54 (20/114) |  | 18.80 (27.63) |  |
|  | Ekali II | 78.81 (119/151) |  | 4023.13 (7733.49) |  | 16.56 (25/151) |  | 20.16 (2138.21) |  |
|  | Ekok | 71.25 (57/80) |  | 2689.96 (5913.93) |  | 10.00 (8/80) |  | 827.00 (769.94) |  |
|  | Essazok | 67.16 (135/201) |  | 2754.96 (3650.20) |  | 13.43 (27/201) |  | 164.74 (4.13) |  |
|  | Kamba | 75.86 (44/58) |  | 3017.45 (8603.15) |  | 10.34 (6/58) |  | 10.67 (17.11) |  |
|  | Koumou | 53.40 (55/103) |  | 3655.85 (5546.48) |  | 17.48 (18/103) |  | 16.89 (6.64) |  |
|  | Mekomba | 72.50 (116/160) |  | 2525.10 (7834.63) |  | 15.00 (24/160) |  | 12.67 (184.01) |  |
|  | Nkassomo | 53.45 (31/58) |  | 3435.35 (8560.84) |  | 18.97 (11/58) |  | 75.64 (8.82) |  |
|  | Nkilzok | 78.38 (87/111) |  | 4710.44 (2946.73) |  | 18.92 (21/111) |  | 13.71 (725.86) |  |
|  | Nkolmefou | 67.36 (97/144) |  | 1806.52 (19086.16) |  | 8.33 (12/144) |  | 223.33 (1437.94) |  |
|  | Nkolnda | 41.76 (114/273) |  | 6936.00 (4679.77) |  | 11.72 (32/273) |  | 273.25 (14.75) |  |

*Densities are expressed as parasites/µl and shown with standard deviation (sd). P_values were obtained using χ^2^ test for prevalence of infection. Wilcoxon sum rank tests were computed for two group comparisons on log-transformed parasite densities and GLMs were used for multiple comparisons. Significant P_values are shown in bold.*

**Table S3** Characteristics of the gametocyte donors and parameters of mosquito infection according to the feeding experiments

| **Characteristics of the gametocyte donor** | | | | | | |  | **Whole blood** | | **OWN blood mixture** | | **AB replacement** | |
| --- | --- | --- | --- | --- | --- | --- | --- | --- | --- | --- | --- | --- | --- |
| **HBB** | **ID** | **Age** | **Sex** | **MOI** | **ABS** | **SBS** |  | **IP (n/N)** | **II (sd)** | **IP (n/N)** | **II (sd)** | **IP (n/N)** | **II (sd)** |
| HbAA |  |  |  |  |  |  |  |  |  |  |  |  |  |
|  | C337 | 8 | M | 5 | 2231 | na |  | 0.39 (9/23) | 4.00 (2.45) | 0.62 (13/21) | 4.85 (4.24) | 0.80 (16/20) | 6.06 (3.32) |
|  | C340 | 12 | F | 2 | 949 | 32 |  | 0.47 (17/36) | 2.18 (1.98) | 0.50 (21/42) | 4.76 (3.67) | 0.63 (38/60) | 6.16 (4.10) |
|  | C366 | 12 | F | na | 0 | 32 |  | 0.48 (15/31) | 2.07 (1.33) | 0.44 (16/36) | 1.75 (0.68) | 0.62 (26/42) | 2.54 (1.68) |
|  | C393 | 8 | F | 3 | 688 | 40 |  | 0.79 (15/19) | 3.87 (2.70) | 0.69 (37/54) | 4.30 (3.75) | 0.69 (33/48) | 4.88 (4.48) |
|  | C409 | 6 | M | 2 | 0 | 144 |  | 0.41 (30/74) | 9.53 (5.59) | 0.24 (20/82) | 7.70 (6.60) | 0.74 (70/94) | 22.77 (15.79) |
|  | C415 | 10 | M | 2 | 768 | 128 |  | 0.61 (44/72) | 5.45 (4.89) | 0.54 (42/78) | 6.95 (6.31) | 0.79 (62/78) | 23.81 (19.15) |
|  | C427 | 13 | F | 3 | 288 | 16 |  | 0.23 (9/39) | 1.22 (0.67) | 0.21 (7/34) | 1.14 (0.38) | 0.45 (17/38) | 1.82 (1.38) |
|  | C431 | 5 | F | 2 | 368 | 296 |  | 0.04 (1/23) | 1.00 (NA) | 0.40 (25/62) | 2.48 (1.78) | 0.70 (14/20) | 5.93 (5.74) |
|  | C432 | 10 | M | 6 | 896 | 16 |  | 0.11 (3/27) | 1.00 (0.00) | 0.73 (19/26) | 6.84 (5.18) | 0.91 (52/57) | 8.56 (5.49) |
|  | C436 | 13 | M | 4 | 128 | 40 |  | 0.44 (16/36) | 1.44 (0.73) | 0.06 (2/33) | 11.50 (14.85) | 0.76 (32/42) | 5.09 (4.66) |
|  | C441 | 5 | M | 2 | 384 | 40 |  | 0.57 (16/28) | 4.25 (4.42) | 0.65 (17/26) | 8.53 (7.11) | 0.96 (24/25) | 9.75 (6.17) |
|  | C451 | 7 | F | 1 | 5808 | 1104 |  | 0.68 (21/31) | 110.86 (79.71) | 0.93 (28/30) | 72.46 (37.68) | 0.96 (26/27) | 57.27 (32.15) |
|  | C457 | 11 | F | 2 | 448 | 88 |  | 0.77 (23/30) | 7.13 (4.52) | 0.56 (18/32) | 2.83 (2.26) | 0.90 (27/30) | 6.07 (4.21) |
|  | C458 | 5 | M | 7 | 2976 | 8 |  | 0.76 (31/41) | 4.77 (3.52) | 0.26 (10/39) | 1.40 (0.70) | 0.86 (48/56) | 5.27 (3.24) |
|  | C512 | 10 | F | 6 | 3344 | 64 |  | 0.50 (12/24) | 3.08 (2.64) | 0.55 (12/22) | 5.50 (3.00) | 0.81 (25/31) | 7.84 (5.23) |
|  | C513 | 9 | F | 7 | 1792 | 88 |  | 0.60 (18/30) | 4.56 (5.09) | 0.24 (11/45) | 2.64 (1.96) | 0.77 (37/48) | 5.65 (4.45) |
|  | C519 | 5 | F | 1 | 0 | 368 |  | 0.50 (19/38) | 17.53 (13.04) | 0.71 (25/35) | 4.52 (4.81) | 0.80 (43/54) | 20.72 (13.62) |
|  | C521 | 9 | F | 8 | 192 | 56 |  | 0.25 (13/53) | 2.08 (1.50) | 0.29 (14/48) | 1.29 (0.83) | 0.58 (29/50) | 2.79 (1.66) |
|  | C535 | 12 | F | 6 | 2624 | 152 |  | 0.97 (68/70) | 33.25 (21.17) | 0.43 (30/69) | 3.23 (2.36) | 0.91 (49/54) | 37.71 (17.14) |
|  | C542 | 8 | F | 5 | 1088 | 56 |  | 0.63 (40/64) | 12.45 (10.40) | 0.78 (49/63) | 7.12 (6.32) | 0.90 (54/60) | 15.44 (9.95) |
|  | C550 | 11 | F | 5 | 0 | 32 |  | 0.48 (20/42) | 7.70 (5.88) | 0.36 (10/28) | 3.00 (2.98) | 0.86 (32/37) | 13.13 (10.10) |
| HbAS |  |  |  |  |  |  |  |  |  |  |  |  |  |
|  | C395 | 6 | F | 3 | 256 | 56 |  | 0.26 (10/39) | 2.90 (2.88) | 0.47 (7/15) | 8.86 (7.36) | 0.94 (31/33) | 9.16 (6.61) |
|  | C402 | 6 | F | 1 | 0 | 200 |  | 0.55 (22/40) | 4.36 (3.44) | 0.57 (16/28) | 4.56 (6.46) | 0.87 (27/31) | 29.96 (17.86) |
|  | C403 | 5 | M | 1 | 0 | 8 |  | 0.27 (6/22) | 2.00 (0.89) | 0.40 (8/20) | 1.63 (0.74) | 0.75 (18/24) | 5.28 (4.60) |
|  | C428 | 13 | F | 4 | 2112 | 48 |  | 0.45 (25/55) | 3.16 (3.76) | 0.46 (19/41) | 2.63 (1.50) | 0.45 (17/38) | 4.06 (3.75) |
|  | C433 | 6 | M | 1 | 208 | 56 |  | 0.60 (33/55) | 3.61 (2.40) | 0.73 (19/26) | 6.84 (5.18) | 0.91 (52/57) | 8.56 (5.49) |
|  | C435 | 7 | M | 2 | 272 | 72 |  | 0.76 (38/50) | 19.24 (13.61) | 0.30 (13/43) | 9.08 (8.79) | 0.97 (28/29) | 17.36 (14.48) |
|  | C445 | 13 | F | 1 | 0 | 96 |  | 0.83 (19/23) | 9.68 (8.06) | 0.64 (38/59) | 4.68 (4.62) | 0.97 (29/30) | 16.31 (8.11) |
|  | C448 | 5 | M | 2 | 8416 | 768 |  | 0.93 (25/27) | 59.04 (42.90) | 0.75 (18/24) | 33.72 (33.77) | 1.00 (31/31) | 65.45 (51.17) |
|  | C455 | 3 | F | 2 | 45984 | 304 |  | 0.67 (20/30) | 4.05 (2.52) | 0.50 (15/30) | 2.93 (2.25) | 0.87 (26/30) | 18.62 (17.82) |
|  | C456 | 3 | M | 2 | 0 | 104 |  | 0.90 (28/31) | 50.29 (27.53) | 0.71 (12/17) | 4.17 (3.54) | 0.97 (30/31) | 34.40 (17.81) |
|  | C515 | 6 | M | 5 | 0 | 688 |  | 0.78 (18/23) | 76.11 (47.18) | 0.77 (49/64) | 44.02 (35.09) | 0.88 (43/49) | 112.58 (66.21) |
|  | C520 | 7 | F | 6 | 11648 | 72 |  | 0.58 (19/33) | 3.84 (3.06) | 0.56 (22/39) | 2.45 (1.90) | 0.79 (33/42) | 6.12 (4.17) |
|  | C544 | 7 | M | 1 | 0 | 32 |  | 0.82 (51/62) | 7.04 (5.09) | 0.34 (11/32) | 4.09 (3.73) | 0.64 (16/25) | 2.31 (1.54) |

***HBB,*** *hemoglobin genotype ;* ***ID****, blood donor code ;* ***MOI****, multiplicity of infection (minimum number of clones within the parasite isolate);* ***ABS****, asexual blood stages (p/µl);* ***SBS****, sexual blood stages (p/µl);* ***IP****, prevalence of infection (percentage of mosquitoes with at least one oocyst developed in the midgut at day 8 post-infection);* ***n/N****, number of infected mosquitoes/number of dissected mosquitoes;* ***II****, infection intensity (mean number of oocysts per midgut);* ***sd****, standard deviation.*

**Table S4** Estimates of the zero-inflated negative binomial generalized linear mixed model for oocyst count in the OWN membrane feeding condition assays

| Count model | |  | |  | |
| --- | --- | --- | --- | --- | --- |
| Predictors | IRR | | CI | | *P*-value |
| (Intercept) | 4.48 | | 2.74 – 7.33 | | <0.001 |
| genotype [HbAS] | 1.51 | | 0.69 – 3.29 | | 0.300 |
| gametocytemia | 2.53 | | 1.76 – 3.64 | | <0.001 |
| MOI | 1.02 | | 0.69 – 1.52 | | 0.912 |
| Zero-inflation model | | | | |  |
| Predictors | OR | | CI | | *P*-value |
| (Intercept) | 0.91 | | 0.55 – 1.52 | | 0.722 |
| genotype [HbAS] | 0.53 | | 0.23 – 1.24 | | 0.143 |
| gametocytemia | 0.75 | | 0.49 – 1.12 | | 0.160 |
| MOI | 0.94 | | 0.61 – 1.43 | | 0.759 |

*Dispersion parameter for truncated_nbinom2 family (): 0.98 ; MOI, multiplicity of infection ; IRR, Incidence Rate Ratio ; OR, Odd Ratio ; CI, confidence interval*

**Table S5** Estimates of the zero-inflated negative binomial generalized linear mixed model for oocyst count in the whole blood membrane feeding assays

| Count model | |  | |  | |
| --- | --- | --- | --- | --- | --- |
| Predictors | IRR | | CI | | *P*-value |
| (Intercept) | 4.48 | | 2.74 – 7.33 | | <0.001 |
| genotype [HbAS] | 1.51 | | 0.69 – 3.29 | | 0.300 |
| gametocytemia | 2.53 | | 1.76 – 3.64 | | <0.001 |
| MOI | 1.02 | | 0.69 – 1.52 | | 0.912 |
| Zero-inflation model | | | | |  |
| Predictors | OR | | CI | | *P*-value |
| (Intercept) | 0.91 | | 0.55 – 1.52 | | 0.722 |
| genotype [HbAS] | 0.53 | | 0.23 – 1.24 | | 0.143 |
| gametocytemia | 0.75 | | 0.49 – 1.12 | | 0.160 |
| MOI | 0.94 | | 0.61 – 1.43 | | 0.759 |

*Dispersion parameter for truncated_nbinom2 family (): 1.49 ; MOI, multiplicity of infection ; IRR, Incidence Rate Ratio ; OR, Odd Ratio ; CI, confidence interval*

**Table S6** Results of the zero-inflated negative binomial generalized linear mixed model for oocyst count in the AB replacement feedings

| Count model | |  | |  | |
| --- | --- | --- | --- | --- | --- |
| Predictors | IRR | | CI | | *P*-value |
| (Intercept) | 7.94 | | 5.10 – 12.37 | | <0.001 |
| genotype [HbAS] | 1.72 | | 0.84 – 3.52 | | 0.140 |
| Zero-inflation model | | | | |  |
| Predictors | OR | | CI | | *P*-value |
| (Intercept) | 0.24 | | 0.16 – 0.36 | | <0.001 |
| genotype [HbAS] | 0.56 | | 0.29 – 1.10 | | 0.093 |

*IRR, Incidence Rate Ratio ; OR, Odd Ratio ; CI, confidence interval*

**Table S7** Results of the zero-inflated negative binomial generalized linear mixed model for oocyst count in the OWN feeding assays

| Count model | |  | |  | |
| --- | --- | --- | --- | --- | --- |
| Predictors | IRR | | CI | | *P*-value |
| (Intercept) | 2.98 | | 1.77 – 5.02 | | <0.001 |
| genotype [HbAS] | 1.53 | | 0.67 – 3.52 | | 0.317 |
| Zero-inflation model | | | | |  |
| Predictors | OR | | CI | | *P*-value |
| (Intercept) | 1.08 | | 0.74 – 1.59 | | 0.687 |
| genotype [HbAS] | 0.73 | | 0.39 – 1.36 | | 0.315 |

*IRR, Incidence Rate Ratio ; OR, Odd Ratio ; CI, confidence interval*

**Table S8** Results of the zero-inflated negative binomial generalized linear mixed model for oocyst count in the whole blood feedings

| Count model | |  | |  | |
| --- | --- | --- | --- | --- | --- |
| Predictors | IRR | | CI | | *P*-value |
| (Intercept) | 3.66 | | 1.97 – 6.81 | | <0.001 |
| genotype [HbAS] | 2.06 | | 0.77 – 5.48 | | 0.149 |
| Zero-inflation model | | | | |  |
| Predictors | OR | | CI | | *P*-value |
| (Intercept) | 0.94 | | 0.59 – 1.52 | | 0.811 |
| genotype [HbAS] | 0.52 | | 0.24 – 1.12 | | 0.095 |

*IRR, Incidence Rate Ratio ; OR, Odd Ratio ; CI, confidence interval*
